# A wide range of chromosome numbers result from unreduced gamete production in *Brassica juncea* × *B*. *napus* (AABC) interspecific hybrids

**DOI:** 10.1038/s41437-024-00738-6

**Published:** 2024-11-30

**Authors:** Charles Addo Nyarko, Elvis Katche, Mariana Báez, Zhenling Lv, Annaliese S. Mason

**Affiliations:** 1 Plant Breeding Department, INRES, University of Bonn, Kirschallee 1, 53115 Bonn, Germany; 2 Plant Breeding Department, Justus Liebig University, Heinrich-Buff-Ring 26-32, 35392 Giessen, Germany

**Keywords:** Polyploidy in plants, Polyploidy in plants, Experimental evolution, Plant hybridization

## Abstract

The establishment of successful interspecies hybrids requires restoration of a stable “2n” chromosome complement which can produce viable “n” gametes. This may occur (rarely) via recombination between non-homologous chromosomes, or more commonly is associated with a doubling of parental chromosome number to produce new homologous pairing partners in the hybrid. The production of unreduced “2n” gametes (gametes with the somatic chromosome number) may therefore be evolutionarily useful by serving as a key pathway for the formation of new polyploid hybrids, as might specific mechanisms permitting recombination between non-homologous chromosomes. Here, we investigated chromosome complements and fertility in third generation interspecific hybrids (AABC) resulting from a cross between allopolyploids *Brassica juncea* (AABB) × *B*. *napus* (AACC) followed by self-pollination for two generations. Chromosome numbers ranged from 2*n* = 48–74 in the experimental population (35 plants), with 9–16 B genome chromosomes and up to 4 copies of A genome chromosomes. Unreduced gamete production leading to a putative genome structure of approximately AAAABBCC was hence predicted to explain the high chromosome numbers observed. Additionally, the estimation of nuclei number in post-meiotic sporads revealed a higher frequency of unreduced gametes (0.04–5.21%) in the third generation AABC interspecific hybrids compared to the parental *Brassica juncea* (0.07%) and *B*. *napus* (0.13%). Our results suggest that unreduced gamete production in the subsequent generations following interspecific hybridization events may play a critical role in restoration of more stable, fertile chromosome complements.

## Introduction

A number of important plant and animal species have been identified as polyploids: organisms whose genomes have more than two complete sets of chromosomes (Madlung 2013). It is now acknowledged that the evolutionary history of all flowering plants includes at least one polyploidisation event (Jiao et al. 2011). Two main forms of polyploidy are commonly recognised: auto-and allo-polyploidy. Autopolyploids occur through genome duplication or hybridization between individuals within the same species, while allopolyploids occur through the hybridization between two different species accompanied by genome doubling (Stebbins 1947). Some well-known allopolyploids include allohexaploid bread wheat (*Triticum aestivum*) and allotetraploid *Brassica napus*, also known as oilseed rape, rapeseed or canola. Although the importance of allopolyploidy for both crop production (Ramanna and Jacobsen 2003) and speciation (Feldman and Levy 2005; Soltis and Soltis 2009) is now well-recognised, several important questions about the actual processes underlying interspecific hybridisation, polyploidisation and species establishment events remain unanswered. Specifically, to what extent these processes of polyploidy and hybridisation are interdependent, and how exactly two species come together to produce new, stable, allopolyploid hybrids, is currently a topic of major interest.

In this study, we aimed to investigate chromosome inheritance and fertility in hybrid progeny between *B. napus* (AACC) and *B. juncea* (AABB). The *Brassica* genus is a well-known system for studies of interspecific hybridisation and polyploidy: six species share a core genetic relationship where three diploid species *B. rapa*, *B. nigra* and *B. oleracea* hybridised to produce allotetraploid species *B. juncea*, *B. napus* and *B. carinata*, with genome complements of AA, BB, CC and AABB, AACC and BBCC respectively. All of these species can be hybridised with each other, with a greater or lesser degree of success (FitzJohn et al. 2007; Katche et al. 2020). So far, the majority of studies involving hybridization between *Brassica* allotetraploid species *B. napus* and *B. juncea* were aimed at making viable, fertile backcross lines in order to introgress useful traits between these two species (Bing et al. 1996; Choudhary and Joshi 2013; Frello et al. 1995; Heenan et al. 2007; Pu et al. 2005; Roy 1980; Tsuda et al. 2011).

A few studies have investigated chromosome pairing and segregation and allele inheritance in AABC hybrids. Regular metaphase I pairing between homologous A-genome chromosomes has been observed in first generation (2n = AABC) F_1_ hybrids (Choudhary and Joshi 2013; Mason et al. 2010), as well as high fidelity (proper segregation of A-genome chromosomes from the two subgenomes) segregation of alleles belonging to the *B. juncea* (A^j^) and *B. napus* (A^n^) subgenomes (Mason et al. 2014a). Non-homologous chromosome pairing between A and C genome chromosomes was commonly observed in the first generation AABC hybrids, as well as less-frequent A-B and B-C chromosome interactions (Mason et al. 2010); unreduced or otherwise abnormal gamete production was also observed at frequencies of approximately 2–6% (Mason et al. 2011), and abnormal meiotic behaviour in the form of unpaired univalent chromosomes (an average of 15.2 univalents) observed at metaphase I in 64% out of 162 pollen mother cells (PMCs) analysed, chromatin bridges, lagging and abnormally dividing chromosome associations has also been observed (Choudhary and Joshi 2013).

The majority of first generation AABC hybrids across different genotype combinations have been found to be unable to set self-pollinated seeds (Katche et al. 2023). Coupled with this, a strong bias towards retention rather than loss of the B and C haploid genomes was observed in the few surviving AABC S_1_ hybrids, despite the presence of a full diploid A genome, suggesting that presence of the B and/or C genomes may be necessary for fertility and viability in these allotetraploid-derived hybrids (Katche et al. 2023). Similarly, difficulties in the extraction of the pure diploid AA component from *Brassica napus* (AACC) variety Darmor indicate that the AA genome may not easily survive without the C genome (Pelé et al. 2017). A single S_1_ plant produced higher numbers of seeds: here, we aimed to investigate chromosome behaviour, inheritance and fertility in these S_2_ generation progeny resulting from *B. juncea* by *B. napus* hybrids, and to subsequently gain insight into the factors contributing to the success of this hybrid lineage and potentially hybridization events in general.

## Materials and methods

### Plant material

Interspecific hybridization by hand-emasculation and bud pollination was undertaken between *B. napus* cultivar “Boomer” and *B. juncea* “JN9-04” (hereafter referred to as “N5” and “J1” respectively) to produce F_1_ interspecific hybrid plants (Mason et al. 2010). Only a few true self-pollinated (S_1_ generation) seeds were produced. The growth and in silico characterisation of the S_0_/F_1_ and S_1_ material (using the Illumina Infinium *Brassica* SNP arrays to confirm true self-pollinated progeny) is described in (Katche et al. 2023). Out of the true self-pollinated progeny, one plant (A-02) when self-pollinated produced 182 seeds, which were germinated and grown under glasshouse conditions in June, 2019 at Justus Liebig University Giessen, along with three seeds from each of the two parent lines N5 and J1 as controls. Most of the S_2_ seeds were flat and abnormally shaped, and only 44 seeds germinated (24%) and survived to flowering. Another true S_1_ hybrid plant (A-01) produced 14 self-pollinated seeds from which only one seed was able to germinate (7%) after sowing. All J1 and N5 parental controls germinated successfully; 3 seeds were sown for each parental control. Neither the experimental population or the control individuals showed any noticeable developmental defects.

### Classical cytogenetics

To assess chromosome pairing and segregation, young floral buds were collected in the morning (between 7 am and 11 am) into a glass tube containing Carnoy’s solution (3:1 ethanol: glacial acetic acid) for 12 h at room temperature and then transferred to 50% ethanol at 4 °C for storage until use. The anthers were squashed and stained in 1% acetocarmine solution (1 g carmine powder in 100 ml glacial acetic acid) on a glass slide using tweezers. The debris was then removed from the solution and a cover slip was placed over the glass slide. Cells were analysed for chromosome pairing and segregation at diakinesis, metaphase I and anaphase using the Zeiss Axio Observer 7 microscope, equipped with Axiocam 305 camera, and the images were analysed using Zeiss Zen v3.4 (Carl Zeiss, Oberkochen, Germany).

To estimate the presence of unreduced gametes, young floral buds from 20 *B. juncea* × *B. napus* AABC S_2_ hybrid plants, one plant from each of the two parental control genotypes J1 and N5, as well as the S_0_/F_1_ and S_1_ parents were used. Briefly, a bud was placed on the glass slide and a drop of 1% acetocarmine solution was added with the aid of a Pasteur pipette. A cover slip was then placed on top of the solution and using the end of the tweezers, the cover slip was pressed until anthers took up the acetocarmine solution and cells are released to preserve structure of the cells and easy identification of number of sporads. The slide was then observed for the number and type of daughter cells present (monads, dyads, triads, tetrads, pentads, hexads and heptads) using 200× or 400× magnification of a Zeiss Axiolab 5 microscope. At least 600 cells were counted for each plant, with a minimum of 300 cells for a single bud. The estimation of unreduced gamete production was based on the formula reported in (Mason et al. 2011), (number of unreduced nuclei in dyads (2) and triads (1)/ (total number of all nuclei in all sporads) × 100.

To evaluate the total chromosome number, root tips from 35 *B. juncea* × *B. napus* AABC S_2_ hybrid plants and one plant from each of the two parental genotypes were used. Following (Snowdon 2007), the young root tips were collected into a glass tube containing 0.04% 8-hydroxyquinoline solution for 2 h in the dark at room temperature and then 2 h at 4 °C. The root tips were then fixed in Carnoy’s solution (3:1 ethanol: glacial acetic acid) for 48 h at room temperature before being transferred to 70% ethanol at −20 °C for storage until ready to be used. Root tips were rinsed for 2 × 10 min in deionized water at room temperature to remove the fixative. They were then incubated in 0.01 M citrate solution for 15 min at room temperature after which the citrate solution was removed using a Pasteur pipette. The root tips were then incubated in 250 µl of enzyme solution (0.25 g of 5% Onozuka R-10 cellulase, 0.05 g of 1% Y23 pectolyase, and 5 ml citrate solution) for 30–40 min at 37 °C. Deionized water was then added to the glass dish to stop the reaction and wash the roots for a minimum of 30 min at room temperature. The meristem root tips were transferred to a glass slide using a Pasteur pipette and excess water was blotted out using a wipe (Kimtech Science). One drop of 3:1 ethanol:acetic acid solution was added to the slide and the root tips were scrambled using a pin into a suspension in the solution to disperse the cells to a width of about 1 cm over the glass slide. The slide was then left to dry and a drop of Vectashield Antifade Mounting Medium with 4′, 6- diamidino-2-phenylindole (DAPI), (H-1200, Vector Laboratories) and a cover slip was added. Slides were then observed at 40× or 100× objective lens magnification of the Leica DMR microscope (Leica Microsystems CMS GmbH, Wetzlar, Germany). The images of the chromosomes were captured using the Leica Application suite software (LAS, version 4.5.0). Contrast optimisation and image sizes were adjusted in Microsoft PowerPoint 2019 (Microsoft Corporation).

### Molecular cytogenetics

#### Karyotyping *B. juncea × B. napus* AABC interspecific hybrids using Fluorescent in situ Hybridisation (FISH) with repetitive DNA sequences and whole genomic DNA (GISH)

Mitotic chromosome cells at metaphase as well as meiotic chromosomes at diakinesis, anaphase I and anaphase II were used for GISH and FISH. *Brassica nigra* whole genomic DNA and *Brassica napus* centromeric repeat mixed probes CentBr1 and CentBr2 (Xiong et al. 2011) were labelled with Alexa Fluor 488-5-dUTP (C11397, Thermo Fisher Scientific) and Texas Red-5-dCTP (NEL426001EA, Perkin Elmer Life Sciences) via the nick translation method. Hybridization was done following Kato et al. (2006). For oligo chromosome painting-FISH using oligonucleotide probes specific to *Brassica* chromosomes A01 and C1, hybridisation was done as described in Montenegro et al. (2022). After hybridisation and washing of the slides, a drop of Vectashield Antifade Mounting Medium with DAPI (H-1200, Vector Laboratories) was added and then covered with a cover slip. Images were captured using the Zeiss Axio Observer 7 microscope equipped with Axiocam 305 camera. Contrast optimisation and image analysis was done using the Zeiss ZEN 3.4. software, Adobe Photoshop Elements software (v. 24.0, Adobe Systems Incorporated), and image sizes were adjusted in Microsoft PowerPoint 2019 (Microsoft Corporation).

### Fertility estimates

Fertility of the hybrid plants was assessed through the estimation of stained viable pollen, and by counting total numbers of self-pollinated seeds. Pollen viability was assessed for the 44 experimental plants plus three plants from each of the two parental control genotypes J1 and N5. Briefly, two fresh flowers per plant were collected and the anthers were stained in a drop of acetocarmine solution on a glass slide. With the help of tweezers, the anthers were dipped into the carmine solution and crushed to release pollen, following which the anther debris was removed. A cover slip was placed over the solution and pollen was counted at 400× total magnification using the Leica DMRE light microscope (Leica Microsystems CMS GmbH). Plump, regularly shaped pollen were assumed to be viable, while shrunken, small and irregularly shaped pollen were assumed to be non-viable. At least 600 pollen grains from each plant were counted, with a minimum of 300 pollen grains for a single flower.

For the evaluation of total seed set, the flowering plants were bagged (covered with microperforated plastic sleeves) to facilitate self-pollination. At maturity the plants were isolated and kept in a drying chamber where irrigation was stopped to enable complete drying of the seeds. The total number of seeds produced per plant were then isolated from the seed pods, cleaned and counted.

### Statistical analyses and figures

Statistical analyses were carried out using R version 4.0.2 (2020-06-22) and RStudio version 2023.06.1 (2023-07-06) (Posit Team 2023; R Core Team 2022). Figures were generated in RStudio and edited with PowerPoint 2019 (Microsoft Corporation). The distribution of data was analysed using Shapiro-Wilk tests and visualized with QQ-plots to help select the appropriate statistical test. Bartlett’s test was used to check for homogeneity of variance. In instances where neither normality nor homogeneity of variance could be assumed, the Kruskal-Wallis rank sum test (a non-parametric one-way analysis of variance test) was used to assess significant differences in fertility between the experimental hybrids and parental controls. Spearman’s rank correlation was used in finding the correlation between pollen viability, seed set and chromosome number while the Wilcoxon rank-sum test was used in assessing significant differences between pollen viability and seed set of selected chromosome groups.

## Results

### Double the expected chromosome complement (2n = AABC) was observed in most individuals

**Fig. 1:**
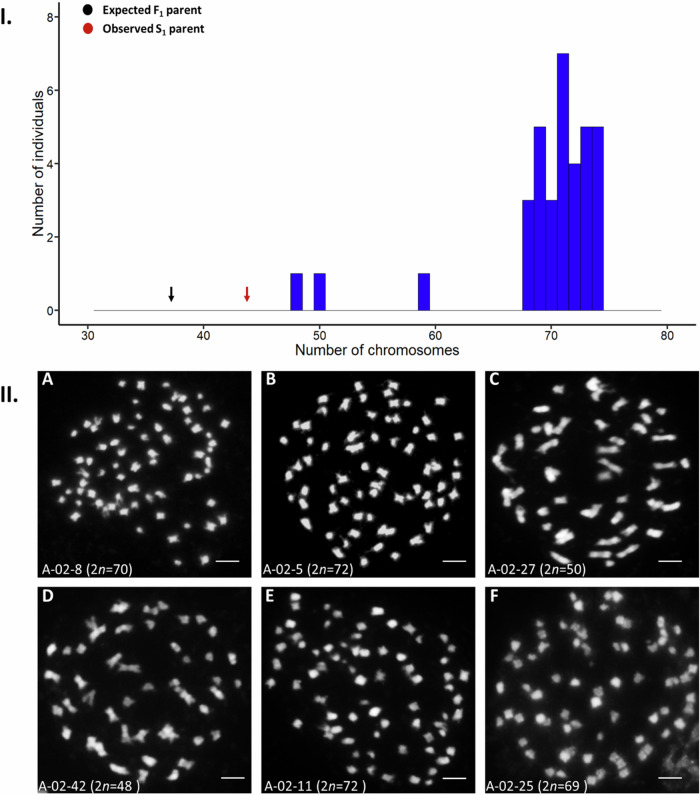
(I) Chromosome numbers (2*n*) in interspecific hybrids resulting from the cross *Brassica juncea* × *B*. *napus* followed by two generations of self-pollination. Predicted chromosome number for a first-generation hybrid is 2*n* = 37 (AABC). Estimated chromosome number for the second-generation (S_1_) parent was 2*n* = 44. The most frequently observed chromosome number in the experimental population was 2*n* = 71. (II) Representative mitotic chromosome spreads in interspecific hybrids resulting from the cross *Brassica juncea × B. napus* followed by two generations of self-pollination. **A** Individual plant A-02-8 with 2*n* = 70 chromosomes. **B** Individual plant A-02-5 with 2*n* = 72 chromosomes. **C** Individual plant A-02-27 with 2n = 50 chromosomes. **D** Individual plant A-02-42 with 2*n* = 48 chromosomes. **E** Individual plant A-02-11 with 2*n* = 72 chromosomes. **F** Individual plant A-02-25 with 2*n* = 69 chromosomes. Chromosomes were counterstained with DAPI, (Gray). Bars = 10 µm.

Mitotic chromosome numbers for 35 S_2_ generation experimental hybrids as well as one plant from each of the parental controls were counted using metaphase spreads prepared from the meristematic root tips and confirmed using fluorescent in situ hybridisation. The parental controls *Brassica juncea* and *B*. *napus* had the expected chromosome numbers 2*n* = 36 and 38 respectively, whereas in the experimental population the modal chromosome number was 2*n* = 71, ranging from 2*n* = 48 to 74 chromosomes. The predicted chromosome number for an F_1_ *Brassica juncea* × *B*. *napus* AABC hybrid is 2*n* = 37, while the doubling of this chromosome complement to AAAABBCC would result in 2*n* = 74 chromosomes. The theoretical maximum chromosome complement which can be derived from self-pollination of an AABC hybrid without the involvement of unreduced gametes or other aberrant meiotic events is 2n = AABBCC = 54 chromosomes. Of the 35 hybrids whose chromosome numbers were estimated, 32 (91%) had a chromosome number ranging from 2*n* = 68–74, one hybrid had a chromosome number of 48, one hybrid had a chromosome number of 50 and one hybrid had a chromosome number of 59 (Fig. 1). Mitotic chromosome counts in the parent plant from the S_1_ generation revealed a chromosome number of 2*n* = 44 (Supplementary Fig. 1).

### Extra copies of chromosomes A01 and C1 confirmed by chromosome-specific oligonucleotide probes (FISH)

**Fig. 2:**
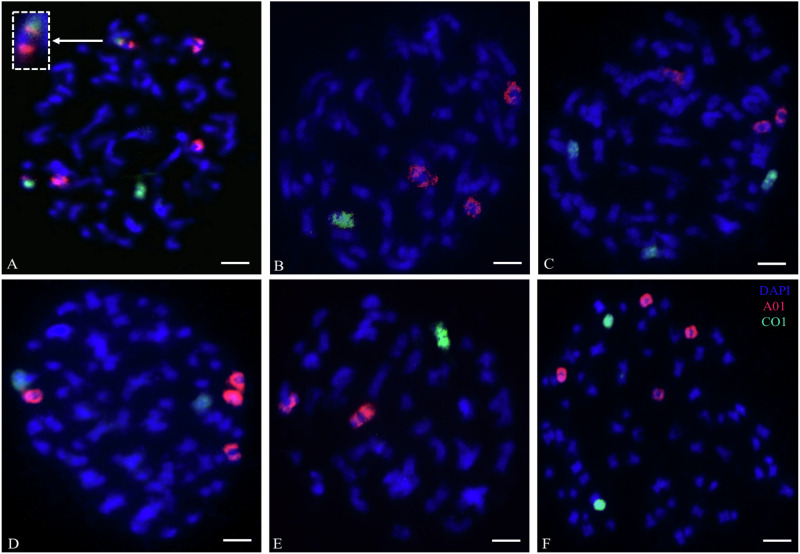
Identification of the number of copies of chromosomes A01 and C1 in interspecific hybrids resulting from the cross *Brassica juncea* × *B. napus* followed by two generations of self-pollination. **A** Individual plant A-02-18 (2*n* = 73) with four A01 chromosomes and two C1 chromosomes including a reciprocal translocation between chromosomes A01 and C1 (inset with a magnified chromosome). **B** Individual plant A-02-39 (2*n* = 59) with three A01 chromosomes and one C1 chromosome. **C** Individual plant A-02-5 (2*n* = 72) with three A01 chromosomes (red) and three C1 chromosomes (green). **D** Individual plant A-02-7 (2*n* = 73) with four A01 chromosomes (red) and two C1 chromosomes (green). **E** Individual plant A-02-27 (2*n* = 50) with two A01 chromosomes and one C1 chromosome. **F** Individual plant A-02-3 (2*n* = 74) with four A01 chromosomes and two C1 chromosomes. Bars = 10 µm.

To confirm the putative genomic structures explaining the observed high and variable chromosome numbers in the *B*. *juncea* × *B*. *napus* S_2_ hybrids (e.g. 2n = AAAABBCC), we used oligo painting FISH with *Brassica* chromosome A01- and C1-specific oligonucleotide probes to identify the number of these respective chromosomes on mitotic spreads in six experimental individuals, as well as in the parental *B*. *juncea* and *B*. *napus* as controls. For the parental control *Brassica juncea* we observed two copies of chromosome A01, and in *B. napus* two copies of chromosome A01 and two copies of chromosome C1, as expected (Supplementary Fig. 2). In sample A-02-18 with 73 chromosomes, we observed the presence of four copies of chromosome A01 and two copies of chromosome C1, including a putative reciprocal translocation between chromosomes A01 and C1 (Fig. 2A). In sample A-02-39 with 59 chromosomes, we observed the presence of three copies of chromosome A01 and one copy of chromosome C1 (Fig. 2B). In sample A-02-05 with 72 chromosomes, we observed three copies for both chromosome A01 and C1 (Fig. 2C). In sample A-02-07 with 73 chromosomes, we observed four chromosome A01 copies and two chromosome C1 copies (Fig. 2D). In sample A-02-27 with 50 chromosomes, we observed two chromosome A01 copies and one chromosome C1 copy (Fig. 2E). In sample A-02-3 with 74 chromosomes, we observed four chromosome A01 copies and two chromosome C1 copies (Fig. 2F). As more than two copies of chromosome A01 cannot be explained without unreduced gamete formation or other aberrant meiotic events, these findings support a genome complement of ~ (2n = AAAABBCC) with accompanying aneuploidy (usually chromosome loss rather than gain) in most individuals.

### Numbers of A/C and B-genome chromosomes in *B. juncea* × *B. napus* S_2_ hybrids

**Fig. 3:**
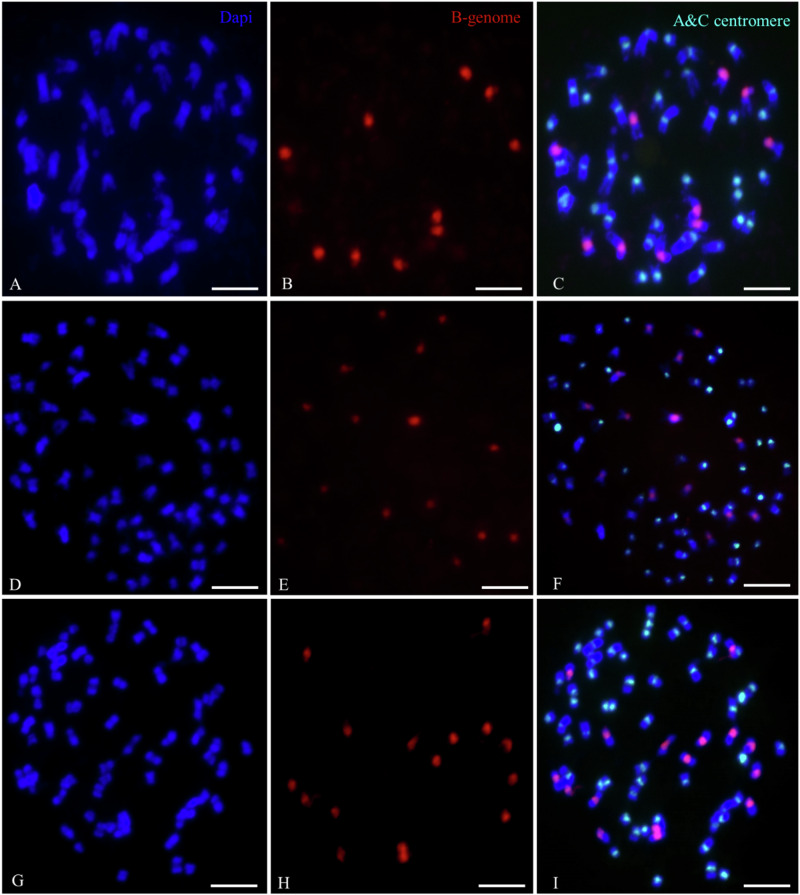
Identifying the B-genome (red), A and C-genome chromosomes (green) in interspecific hybrids resulting from the cross *Brassica juncea* × *B. napus* followed by two generations of self-pollination. **A**–**C** Plant A-02-27 with ten B-genome chromosomes and 40 A/C genome chromosomes. **D**–**F** Plant A-02-3 with 16 B-genome chromosomes and 58 A/C genome chromosomes. **G**–**I** Plant A-02-05 with 16 B-genome chromosomes and 56 A/C genome chromosomes. Chromosomes were counterstained with DAPI (blue). Bars = 10 µm.

**Fig. 4:**
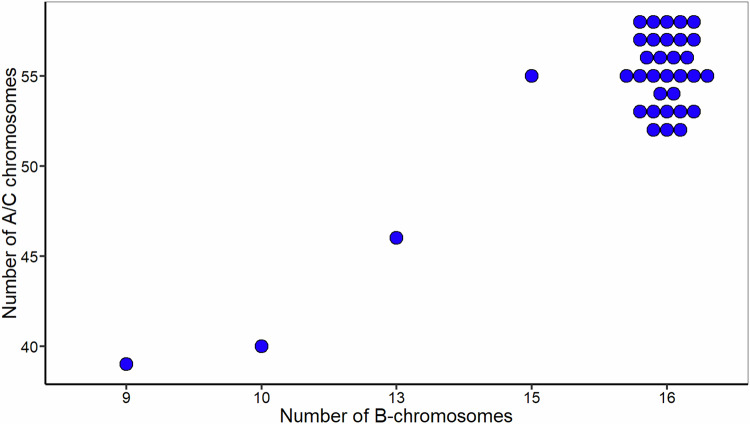
Distribution of the number of B and A/C-genome chromosomes in interspecific hybrids resulting from the cross *Brassica juncea* × *B. napus* followed by two generations of self-pollination. The modal B and A/C genome chromosome numbers were 16 and 55 respectively.

In addition to the identification of specific chromosomes using oligo painting-FISH, we estimated the number of *Brassica* B, A and C genome chromosomes in mitotic chromosome spreads in all 35 experimental individuals using whole genomic DNA from *Brassica nigra* (to label the B genome chromosomes) and centromeric tandem repeat probes CentBr1 and CentBr2, which together label all A- and C-genome chromosomes (Xiong et al. 2011), although do not distinguish between them (Fig. 3). This specific hybridization of the probe sets was confirmed using the parental *B*. *juncea* cv. “J1”and *B*. *napus* cv. “N5” (Supplementary Fig. 3). Most individuals (31, 88%) had 16 B chromosomes, and 9, 10, 13, and 15 B-genome chromosomes were each represented in a single individual (Fig. 4). Similarly, for the A- and C-genome chromosomes, we observed chromosome numbers ranging from 2*n* = 39–58 (Fig. 4). Most individuals (32, 91%) had 52–58 A- and C-genome chromosomes, whereas three individuals (9%) had 39–46 A- and C-genome chromosomes. The most frequently observed A/C genome chromosome number was 55.

### Varying frequencies of male unreduced gametes (estimated via sporad observation) in the *B. juncea* × *B. napus* S_2_ hybrids

**Fig. 5:**
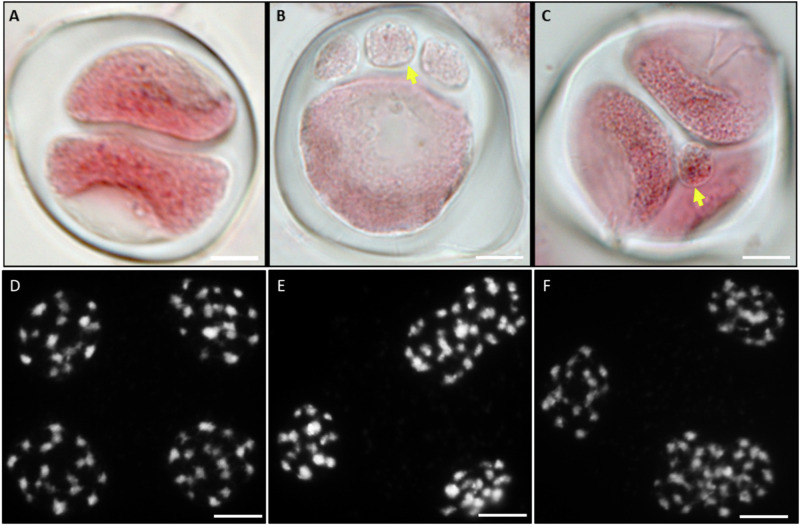
Representative sporad types observed in the experimental hybrids resulting from the cross *Brassica juncea* × *B*. *napus* followed by two generations of self-pollination*.* **A** Dyad, (**B**) unbalanced tetrad with one large and three small nuclei (yellow arrows), (**C**) tetrad with an additional micronucleus, (**D**) representative meiotic behaviour at Anaphase II in the *B*. *napus* parental genotype “N5” showing equal segregation of chromosomes, (**E**, **F**) Anaphase II showing putative triads with unequally segregated chromosomes. Bars = 10 µm.

Young floral buds at the sporad stage (post-meiosis but before cytokinesis) were used to estimate the production of unreduced gametes based on the number of nuclei present in each sporad: monads, dyads, triads, tetrads, pentads and hexads. As well as a high frequency of tetrads, dyads (Fig. 5A) were also frequently observed, and small “micronuclei” structures were occasionally detected (Fig. 5B, C). As expected, the product of male meiosis in the parental control genotype *Brassica napus* cv. “Boomer” was almost entirely tetrads (Fig. 5D). Occasional triads (Fig. 5E, F) were also observed in 4 out of 414 (1.0%) pollen mother cells. We estimated the frequency of unreduced gametes following (Mason et al. 2011) with the following modification, assuming dyads form two unreduced gametes, triads form one unreduced gamete and two reduced gametes while tetrads form four normal, reduced gametes. Unreduced gamete rate was then estimated based on the formula (number of unreduced nuclei in dyads (2) and triads (1)/(total number of all nuclei in all sporads) × 100. We observed that the *B. juncea* and *B. napus* parental genotypes had a lower rate of unreduced gametes (0.07% unreduced gametes in *B. juncea* and 0.13% in *B. napus*) compared to the experimental interspecific hybrids which produced unreduced gametes ranging from 0.04 to 5.21% (286 unreduced nuclei out of 52,320 total nuclei (0.55%)). The S_0_/F_1_ and S_1_ parents recorded 5.75% unreduced gametes (250 unreduced nuclei out of 4349 total nuclei), and 0.04% unreduced gametes (2 unreduced nuclei out of 4667).

#### Meiotic chromosome pairing and segregation between the B and A/C sub-genomes of *Brassica juncea* × *B*. *napus*

**Fig. 6:**
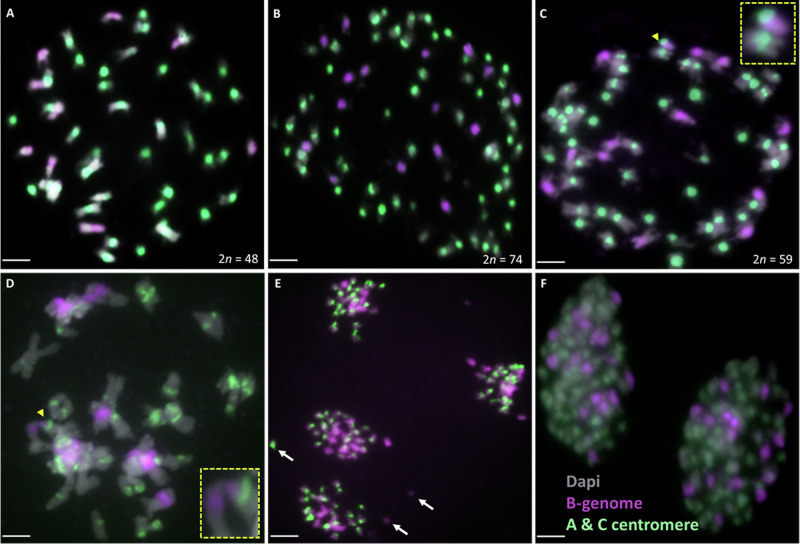
Karyotype instability in the interspecific hybrids resulting from the cross *Brassica juncea* × *B*. *napus* followed by two generations of self-pollination*.* Fluorescent in situ hybridisation (FISH) of mitotic and meiotic chromosome spreads with B-genome genomic probe (purple) and A/C genome centromeric probe (green). **A**–**C** Mitotic chromosome showing chromosome number variation in the experimental hybrids. **A** Plant A-02-42 with nine B-genome chromosomes and 39 A/C genome chromosomes. **B** Plant A-02-44 with 16 B-genome chromosomes and 58 A/C genome chromosomes. **C** Plant A-02-39 with 13 B-genome chromosomes and 46 A/C genome chromosomes including a translocation involving chromosomes from the B and A/C genomes (inset with magnified chromosomes, yellow). **D** Abnormal meiotic behaviour in the form of a non-homologous chromosome association (yellow arrow, inset with an increased size chromosome in yellow) at diakinesis. **E** Lagging chromosomes (white arrows) at Anaphase II in the S_2_ hybrids. **F** Separation of sister chromatids instead of homologous chromosomes at Anaphase I in First Division Restitution-type 2*n* gamete formation. 16 B-genome chromatids. Bar = 10 µm.

Based on the observed mitotic karyotype variation (Fig. 6A–C) and chromosome introgressions (Fig. 6C), we hypothesized that aberrant meiosis was likely to have occurred. We therefore examined chromosome pairing and segregation between the B and A/C subgenomes in the experimental hybrids during diakinesis (Fig. 6D), metaphase I, and anaphase I and II (Fig. 6E, F) using fluorescent in situ hybridisation with the A/C centromere probes CentBr1 and CentBr2, and whole genomic DNA from *Brassica nigra*. We observed several meiotic abnormalities, including frequent univalents at metaphase I in 93% of PMCs, (*n* = 76), lagging chromosomes at anaphase I and II in 31% of PMCs (*n* = 189) (Fig. 6E) compared to 6% in *B*. *napus* “N5” control (*n* = 32), and occasionally (8.6%), a clearly unequal set of chromosomes at each pole at anaphase I (*n* = 176). For the B-genome chromosomes we observed mostly bivalents (7.3 II) in three individuals at diakinesis, with frequent (65%) non-homologous pairing between the B and A/C genomes (*n* = 37) (Fig. 6D).

### Most lines were fertile, but fertility was not correlated to chromosome number

#### Self-pollinated seed set

**Fig. 7:**
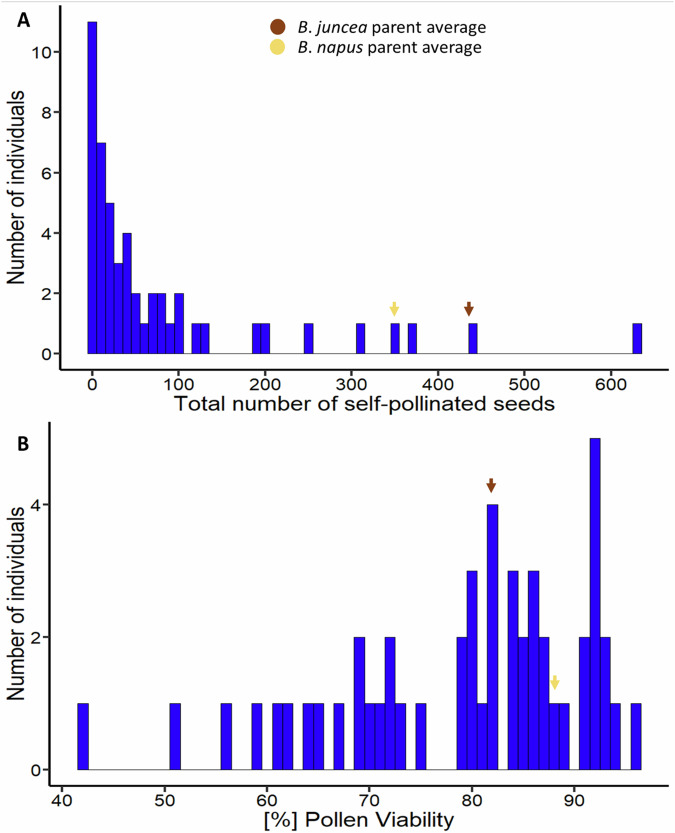
Fertility assessment in interspecific hybrids produced from the cross *Brassica juncea* × *B. napus* followed by two rounds of self-pollination compared to parental controls. **A** Total number of self-pollinated seeds produced per hybrid plant. Arrows indicate average parental values (dark brown: *B. juncea* parent; light brown: *B. napus* parent). Significant differences were observed between the experimental hybrids and the two parental controls (Kruskal-Wallis test, *p* < 0.001). **B** Pollen viability per hybrid plant. Arrows indicate average parental values (dark brown: *B. juncea* parent; light brown: *B. napus* parent). There were no significant differences (*p* > 0.05) in pollen fertility between the AABC hybrids and either the *B*. *napus* or *B*. *juncea* parental controls.

The 44 experimental hybrids had a significantly lower average seed set (42, ranging from 0–203) compared to the parental controls. The *Brassica juncea* “J1” parental control had an average of 438 seeds/plant while the *B. napus* “N5” parental control had an average of 345 seeds/plant (Fig. 7A). There was a significant difference (Kruskal-Wallis test, *p*-value < 0.05) between the experimental hybrids and the two parental controls. Estimation of correlation between the 35 individuals whose chromosome numbers were counted and their respective seed set showed no significant association (Spearman’s rank correlation, *p*-value = 0.55).

#### Pollen viability

The observed pollen varied in shape, ranging from plump, regular shaped pollen that were recorded as viable, to the shrunken, small and irregularly-shaped pollen that were recorded as non-viable. The three *B. juncea* parent plants (J1) had an average pollen viability of 82% while that of the three *B. napus* parental controls (N5) was 88%. The S_2_ hybrid plants had an average pollen viability of 78%, ranging from 42–96% (Fig. 7B). No significant differences in pollen fertility between the AABC S_2_ hybrids and either the *B. napus* or *B. juncea* parental controls were observed (Kruskal-Wallis rank sum test; *p* = 0.2668). Estimation of correlation between the 35 individuals whose chromosome numbers were counted and their corresponding percentage pollen viability showed no significant correlation (Spearman’s rank correlation, *p*-value = 0.52).

## Discussion

In this study, we assessed chromosome inheritance and behaviour and fertility in third generation interspecific hybrids produced from a cross between *B. napus* (2n = AACC = 38) and *B*. *juncea* (2n = AABB = 36) followed by two generations of self-pollination using a combination of classical and molecular cytogenetic techniques, as well as fertility measurements. Unexpectedly high chromosome numbers, ranging from 2*n* = 48 to 2*n* = 74, were recorded for the experimental hybrids. Unreduced gametes were hence assumed to have been produced in the previous generation in order to explain the observed chromosome numbers. This was supported by observations of up to four copies of chromosome A01 and two copies of chromosome C1 as well as the presence of 16 B genome chromosomes, consistent with a 2*n* = AAAABBCC = ~74 chromosome complement. Many of the experimental plants were fertile with an average seed set of 42 seeds per plant. Although no significant correlations were observed between chromosome numbers and fertility in the plants we examined, we assume that unreduced gametes were necessary to restore viability and fertility in this lineage, based on both the fact that the majority of progeny had approximately 2*n* = AAAABBCC chromosome complements, and that previous studies have found most AABC hybrids to be sterile (Katche et al. 2023). Our results suggest that hybridization between young allotetraploid species with one shared subgenome and one divergent subgenome (with minimal homoeologous pairing between the divergent subgenomes) may require an additional round of ploidy elevation to establish a viable, fertile lineage.

In addition to the much higher than expected average chromosome numbers, most experimental individuals were also predicted to be aneuploid. A high rate of aneuploidy with a bias towards the accumulation of extra chromosomes was observed in resynthesized *Brassica napus* allopolyploids (Xiong et al. 2011) and rice segmental allotetraploid populations (Wu et al. 2018). Additionally, in resynthesized allohexaploid wheat, transgenerational (from S_1_ to >S_20_) whole chromosome number variation was also discovered at variable frequencies (Zhang et al. 2013). Aneuploidy therefore appears to be a reoccurring process associated with genomes of nascent polyploid species. How the evolution of a newly formed polyploid genome is impacted by aneuploidy has been examined in various studies (Ge et al. 2009; Jong et al. 2018; Mestiri et al. 2010). Factors from the initial hybridization process and abnormalities in meiotic chromosome pairing or segregation (Chester et al. 2012) have also been implicated as causes of such variation in karyotype instability. In most neo-allopolyploids, meiotic behaviour is usually characterized by irregularities such as multivalent chromosome association as well as by the production of unbalanced gametes, which generates a high frequency of aneuploids, pseudoeuploids (with dosage-compensated aneuploidy between homoeologous chromosome sets) and genotypes with non-homologous recombination (Ramsey and Schemske 2002). Based on the observed chromosome numbers in the third generation, and presence of micronuclei which signify the final steps of complete chromosome elimination (Gernand et al. 2006), as well as the higher rate of unreduced gametes that was observed in the first generation AABC hybrids, we would expect to see some extent of aneuploidy transmitted to the first selfing progeny generation. There is evidence to suggest that aneuploidy and chromosomal abnormalities can persist across multiple generations in nascent polyploids (Chester et al. 2012; Gao et al. 2016; Szadkowski et al. 2010; Xiong et al. 2011); this finding was also supported by our results.

The observation of chromosomal configurations such as multivalent during meiotic analysis signifies the lack of a strict genetic control of paring between chromosomes in these hybrids. This can be attributed to either homologous (A-A) pairing disruption, or the presence of multiple genomes with a high degree of homoeology in the same nucleus (Mason et al. 2010). Although most A genome chromosomes do pair homologously in this hybrid type (98.5%) (Mason et al. 2010), autosyndetic and allosyndetic associations are extremely frequent, with an average of 2.3 A-C chromosome associations (mostly A-A-C trivalents) per PMC. Genetic variation was also found to influence the frequency of allosyndesis between three *B. napus* × *B. juncea* genotypes (Mason et al. 2010). While these homoeologous exchanges can generate genetic diversity and novel phenotypes (Gaeta and Pires 2010; Schiessl et al. 2019; Schranz and Osborn 2004), they may also disrupt the karyotype and lead to erratic meiotic behaviour, aneuploidy and a reduction in fertility (Gaeta and Pires 2010; Pelé et al. 2018), as well as chromosomal variation in the next generations (Chester et al. 2015; Mason and Wendel 2020; Mwathi et al. 2019).

The rate of male unreduced gametes in the experimental population was examined by recording the number of daughter cells present within sporads: monads, dyads, triads, tetrads, pentads and hexads. We discovered that 2*n* gametes were generated in the range (0.04–5.21%) compared to parental controls (0.07% in *B. juncea* and 0.13% in *B. napus*). In addition, we observed a higher rate (5.75%) of unreduced gametes in the S_0_/F_1_ parent, whereas in the S_1_ parent the rate of 2*n* gametes was 0.04%. The observed differences in average rate of unreduced gamete production between the interspecific hybrids and their parental controls is consistent with observations made by (Mason et al. 2014b; Mason et al. 2011; Ramsey and Schemske 1998) who also discovered that unreduced gametes were produced at a higher frequency in interspecific hybrids than in their parents. Mason et al. (2014b) observed an average of 1.9% dyads in 58 allohexaploid individuals with a single hybrid plant that produced 36.2% dyads. Unreduced gamete production was also found to vary greatly between and within 24 species of Brassicaceae (Kreiner et al. 2017). The low frequency of unreduced gametes in the parental genotypes is expected in relatively established species, including allopolyploids that show diploid-like meiotic behaviour (Leitch and Leitch 2008). Also, information on heritable and selectable genetic variation for unreduced gamete production has been discussed in (Brownfield and Köhler 2011; Kreiner et al. 2017; Mason and Pires 2015; Ramsey and Schemske 1998, and references therein), thus, it is possible that genetic factors that were associated with the high 2*n* gamete production in the parental F_1_ AABC plant (e.g. affecting frequency of parallel spindle formation; (Mason et al. 2011; reviewed by De Storme and Mason 2014)) may have been passed on through the progenies.

It seems probable that fertilisation between gametes with different ploidy levels could lead to the observed genome structure in our experimental population, although we could only estimate the frequency of male 2*n* gametes and not female 2*n* gametes (ovules) across the three generations. Various meiotic abnormalities that could lead to 2*n* gamete formation include abnormal cytokinesis, omission of the first or second division and abnormal spindle orientation (Lukaszewski 2022; Oleszczuk and Lukaszewski 2014), with parallel or fused spindles at the second meiotic division being the most common (d’Erfurth et al. 2008). Which of these mechanisms was operative in our population is unknown, but parallel spindles have previously been observed in *Brassica* (*B. napus* by *B. carinata*) interspecific hybrids with CCAB genome complements (Mason et al. 2011), and would explain our observed chromosome karyotype results. Genetic factors, (Clot et al. 2024; d’Erfurth et al. 2008; Harlan and deWet 1975) are also known to affect 2*n* gamete production: in *Arabidopsis thaliana*, the frequency of 2*n* gametes was found to be influenced by multiple genes with *AtPS1* (*Arabidopsis thaliana Parallel Spindle 1*) acting as the major gene: mutation of *AtPS1* led to the formation of a high rate of 2*n* male gametes, 2*n* pollen and triploid plants in the next generation (d’Erfurth et al. 2008). Recently, multiple QTLs regulating unreduced pollen production were also identified in diploid *Solanum tuberosum* (Clot et al. 2024). Of these QTLs, two with major effect co-localised with genes sharing homology with *A*. *thaliana JASON* (a known regulator of spindle orientation).

The 44 S_2_ experimental hybrids had a significantly lower average seed set (42, ranging from 0 - 203) than the average of parental *Brassica juncea* (438 seeds/plant) and *B. napus* (345 seeds/plant) controls. However, only two S_2_ individuals were completely sterile, whereas in both S_1_ and F_1_ generations most individuals were completely or nearly sterile (Katche et al. 2023). Low fertility is often observed in first generation interspecific hybrids in *Brassica* (Gaebelein et al. 2019; Mwathi et al. 2019) as well as other de novo allopolyploids such as synthetic *Arabidopsis suecica* (Chéron et al. 2024) and triticale (Shkutina and Khvostova 1971). Low fertility is thought to be common in newly formed polyploids and interspecific hybrids (reviewed by Ramsey and Schemske 1998) and to directly result from meiotic instability (reviewed by Pelé et al. 2018; but see Srikant et al. 2024 for an alternative explanation in autopolyploid *A. arenosa*). Putatively, unreduced gamete formation leading to the inheritance of complete chromosome complements could be responsible for the few S_1_ plants that generated seeds. The observed chromosome complements in the S_2_ population (Supplementary Table 1) supported a putative AAAABBCC genome structure for at least 32/35 individuals (chromosome numbers 2*n* = 60–74), while two individuals had chromosome numbers of 48 and 50 (likely ~AAABCC) and the last individual also appeared to result from the union of an unreduced gamete and a reduced gamete (three copies of chromosome A01, 2*n* = 59), but with substantial chromosome loss. The fertility estimates (total seed number and percentage pollen viability) from these two chromosome number categories were not significantly different (Wilcoxon rank-sum test, *p* > 0.05). The 32 individuals with chromosome numbers ranging from 2*n* = 60–74 averaged 32 self-pollinated seeds per plant and 77% pollen viability. The three individuals with chromosome numbers 2*n* = 48, 50 and 59 recorded 3, 48 and 131 self-pollinated seeds each, and a percentage pollen viability of 69%, 94% and 71%. Possibly, the lack of correlation between fertility and karyotype in our study could be as a result of the buffering these hybrid types enjoy from the presence of all three genomes, which therefore permits high frequencies of chromosome rearrangements, duplications, and deletions (Mason et al. 2014b). Selection for more viable karyotypes (with more chromosomes) may also have assisted in restoring fertility in these lines.

## Conclusions

We observed a high variation in the number of chromosomes in third-generation AABC hybrids, which we were able to attribute to the involvement of unreduced gametes. As this hybrid type was either sterile or showed very poor fertility in the first generations, this suggests that unreduced gamete production contributed to restoration of more stable, fertile chromosome complements. In conclusion, the chromosome composition, inheritance and fertility of interspecific hybrids between *Brassica juncea* × *Brassica napus* (AABC) described here contributes to our understanding of the successful formation/establishment of hybrid lineages by highlighting the importance of unreduced gametes in generating higher ploidy progeny and hence restoring viability and fertility in interspecific crosses.

### Data archiving

All data generated is available in the manuscript and supplementary files.

## Supplementary information

Supplemental Information
