# Supplementary material for: A wide range of chromosome numbers result from unreduced gamete production in Brassica juncea × B. napus (AABC) interspecific hybrids
Source: Heredity (Edinb). 2024 Nov 30;134(2):98–108. doi: 10.1038/s41437-024-00738-6 (PMC11799209; doi:10.1038/s41437-024-00738-6)
Supplement: Supplementary file 1 — Supplemental Information [file 41437_2024_738_MOESM1_ESM.pdf]

A wide range of chromosome numbers result from unreduced gamete production in *Brassica juncea* × *B. napus* (AABC) interspecific hybrids

Charles Addo Nyarko[1,2], Elvis Katche[2], Mariana Baez[1], Zhenling Lv[1,2], Annaliese S. Mason[1,2,*]

[1] Plant Breeding Department, University of Bonn, Kirschallee 1, 53115, Bonn, Germany

[2] Plant Breeding Department, Justus Liebig University, Heinrich-Buff-Ring 26-32, 35392, Giessen, Germany

*corresponding author: annaliese.mason@uni-bonn.de

Supplemental Information

Supplementary Figure 1: Mitotic metaphase chromosome spreads from the S_1 parental hybrid resulting from the cross *Brassica juncea* × *B. napus* followed by one generation of self-pollination. Predicted chromosome number for a first-generation interspecific hybrid is 2*n* = 37 (AABC) chromosomes. Bar = 10 µm.

Supplementary Figure 2: Identification of the number of copies of chromosomes A01 and C1 in the interspecific hybrids resulting from the cross *Brassica juncea* × *B. napus* followed by two generations of self-pollination. A) Parental *Brassica napus* (2*n* = 38) with 2 A01 chromosomes (red) and 2 C1 chromosomes (Turquoise). **B)** Parental *Brassica juncea* (2*n* = 36) with 2 A01 chromosomes. Chromosomes are counterstained with DAPI (blue). Bars = 10µm.

Supplementary Figure 3: Identification of the A and C-genome chromosomes (green) with *Brassica* centromere probes CentBr1 and CentBr2 in the parental *Brassica juncea* (A) and *B. napus* (B). Chromosomes are counterstained with DAPI (blue). Bars = 10µm.

Supplementary Figure 4: Distribution of the number of B and AC-genome chromosomes in the interspecific hybrids resulting from the cross *Brassica juncea* × *B. napus* followed by two generations of self-pollination.

Supplementary Table 1: Estimated chromosome number, number of copies for Chromosomes A01 and C1, and fertility (total seed set and percentage pollen viability) in the interspecific hybrids resulting from the cross *Brassica juncea* × *B. napus* followed by two generations of self-pollination.

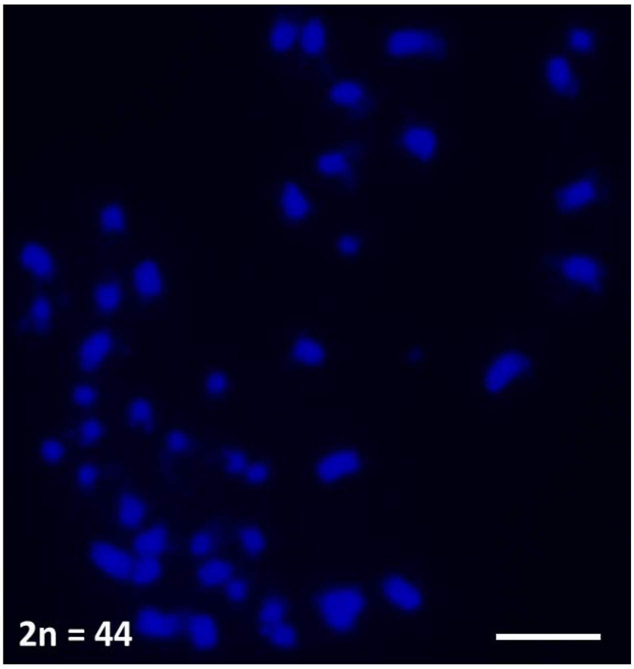

Supplementary Figure 1: Mitotic metaphase chromosome spreads from the S_1 parental hybrid resulting from the cross *Brassica juncea* × *B*. *napus* followed by one generation of self-pollination. Predicted chromosome number for a first-generation interspecific hybrid is 2*n* = 37 (AABC) chromosomes. Bar = 10 µm.

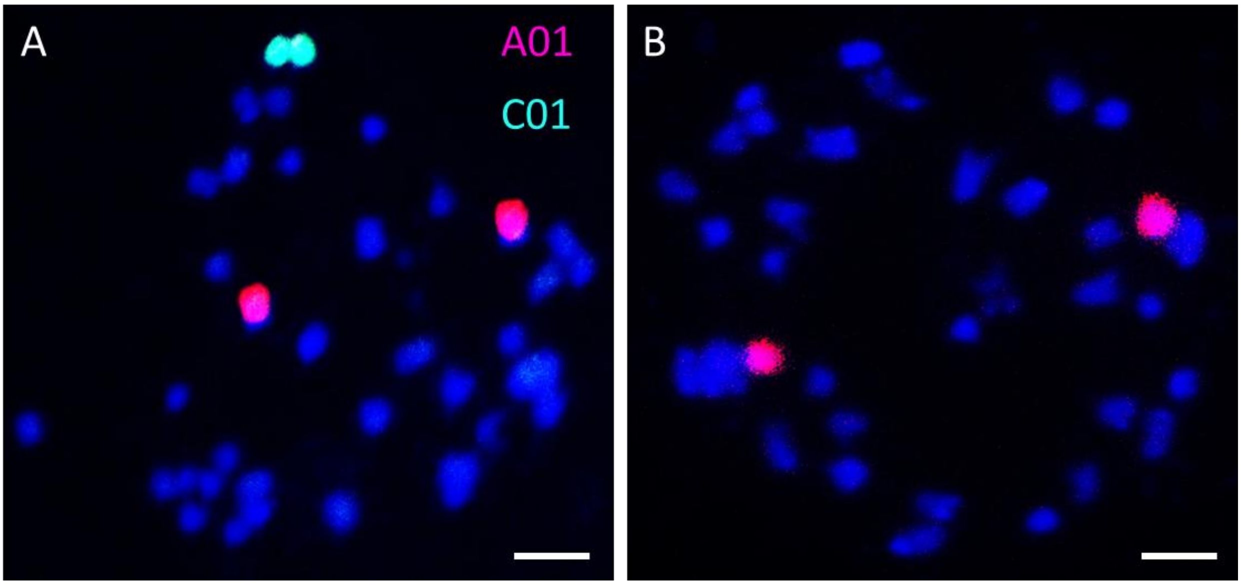

Supplementary Figure 2: Identification of the number of copies of chromosomes A01 and C1 in the interspecific hybrids resulting from the cross *Brassica juncea* × *B. napus* followed by two generations of self-pollination. A) Parental *Brassica napus* with 2 A01 chromosomes (red) and 2 C1 chromosomes (Turquoise). **B)** Parental *Brassica juncea* with 2 A01 chromosomes. Chromosomes are counterstained with DAPI (blue). Bars = 10µm.

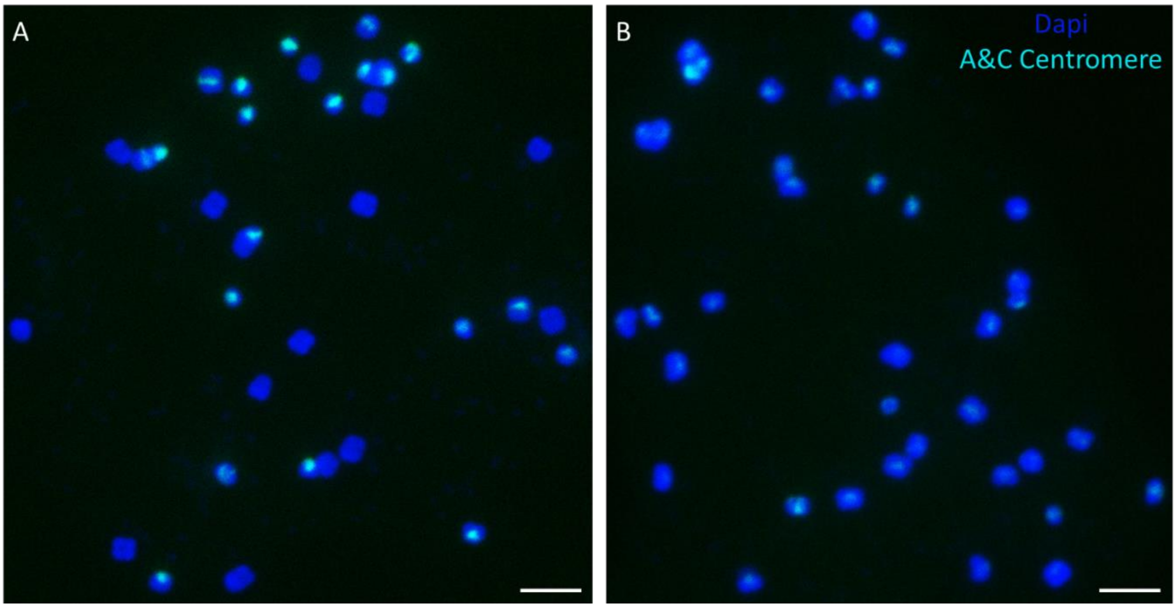

Supplementary Figure 3: Identification of the A and C-genome chromosomes (green) with *Brassica* centromere probes CentBr1 and CentBr2 in the parental *Brassica juncea* (A) and *B*. *napus* (B). Chromosomes are counterstained with DAPI (blue). Bars = 10µm.

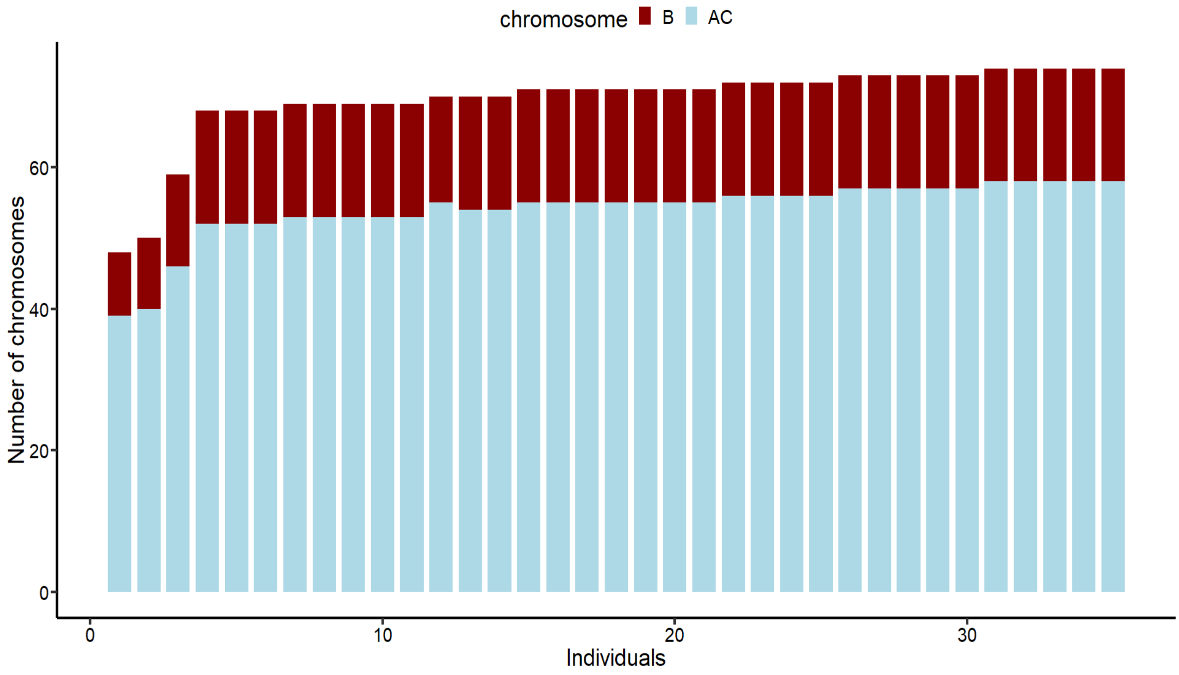

Supplementary Figure 4: Distribution of the number of B and AC-genome chromosomes in the interspecific hybrids resulting from the cross *Brassica juncea* × *B. napus* followed by two generations of self-pollination.

Supplementary Table 1: Estimated chromosome number, number of copies for Chromosomes A01 and C1, and fertility (total seed set and percentage pollen viability) in the interspecific hybrids resulting from the cross *Brassica juncea* × *B. napus* followed by two generations of self-pollination.

Plant	Chromosome A01	Chromosome C1	Chromosome Number	Total Seedset	Pollen Viability (%)
A-02-1			71	41	75
A-02-3	4	2	74	18	64
A-02-4	4	2	73	191	62
A-02-5	3	3	72	7	86
A-02-6			72	35	91
A-02-7	4	2	73	1	84
A-02-8			70	11	92
A-02-10			70	4	51
A-02-11			72	22	96
A-02-12			74	0	67
A-02-13			72	12	92
A-02-14			69	60	82
A-02-15			71	104	72
A-02-16			68	23	73
A-02-17			69	5	85
A-02-18	4	2	73	4	84
A-02-19			68	0	69
A-02-20			71	16	59
A-02-21			69	4	72
A-02-22			71	1	82
A-02-23			68	6	77
A-02-24			73	5	82
A-02-25			69	48	56
A-02-27	2	1	50	48	94
A-02-28			69	88	65
A-02-29			74	2	42
A-02-30			74	11	70
A-02-31			71	82	80
A-02-32			71	41	81
A-02-33			70	22	88
A-02-34			73	77	79
A-02-37			71	36	84
A-02-39	3	1	59	131	71
A-02-42			48	3	69
A-02-44			74	71	91
Brassica napus cv. Boomer_1	2	2	38	312	85
Brassica napus cv. Boomer_2				374	86
Brassica napus cv. Boomer_3				349	92
Brassica juncea cv. J1_1	2		36	631	92
Brassica juncea cv. J1_2				438	93
Brassica juncea cv. J1_3				246	61
